# Arrhythmogenic Cardiomyopathy and Biomarkers: A Promising Perspective?

**DOI:** 10.3390/jcm14197046

**Published:** 2025-10-05

**Authors:** Federico Barocelli, Nicolò Pasini, Alberto Bettella, Antonio Crocamo, Enrico Ambrosini, Filippo Luca Gurgoglione, Eleonora Canu, Laura Torlai Triglia, Francesca Russo, Angela Guidorossi, Francesca Maria Notarangelo, Domenico Corradi, Antonio Percesepe, Giampaolo Niccoli

**Affiliations:** 1 Cardiology Division, Parma University Hospital, 43126 Parma, Italy; nicolo.pasini@unipr.it (N.P.); alberto.bettella@unipr.it (A.B.); acrocamo@ao.pr.it (A.C.); filippolucagurgoglione@gmail.com (F.L.G.); eleonora.canu@unipr.it (E.C.); laura.torlait@gmail.com (L.T.T.); russofr@ao.pr.it (F.R.); aguidorossi@ao.pr.it (A.G.); notarangelof@ao.pr.it (F.M.N.); giampaolo.niccoli@unipr.it (G.N.); 2 Medical Genetics Unit, University Hospital of Parma, 43126 Parma, Italy; eambrosini@ao.pr.it (E.A.); antonio.percesepe@unipr.it (A.P.); 3 Department of Medicine and Surgery, Anatomic Pathology, University of Parma, 43126 Parma, Italy; domenico.corradi@unipr.it

**Keywords:** arrhythmogenic cardiomyopathy, biomarkers, early diagnosis, miRNA, ventricular arrhythmias, sudden cardiac death

## Abstract

Arrhythmogenic cardiomyopathy (ACM; MIM #107970) is a primitive heart muscle disease characterized by progressive myocardial loss and fibrosis or fibrofatty replacement, predisposing patients to ventricular arrhythmias, sudden cardiac death, and heart failure. Despite advances in imaging and genetics, early diagnosis remains challenging due to incomplete penetrance, variable phenotypic expressivity, and the fact that fatal arrhythmic events may often occur in the early stages of the disease. In this context, the identification of reliable biomarkers could enhance diagnostic accuracy, support risk stratification, and guide clinical management. This narrative review examines the current landscape of potential and emerging biomarkers in ACM, including troponins, natriuretic peptides, inflammatory proteins, microRNAs, fibrosis-related markers, and other molecules. Several of these biomarkers have demonstrated associations with disease severity, arrhythmic burden, or structural progression, although their routine clinical utility remains limited. The increasing relevance of genetic testing and non-invasive tissue characterization—particularly through cardiac imaging techniques—should also be emphasized as part of a multimodal diagnostic strategy in which biomarkers may play a complementary role. Although no single biomarker currently meets the criteria for a standalone diagnostic application, ongoing research into multi-marker panels and novel molecular targets offers promising perspectives. In conclusion, the integration of circulating biomarkers with imaging findings, genetic data, and clinical parameters may open new avenues for improving early detection and supporting personalized therapeutic strategies in patients with suspected ACM.

## 1. Introduction

Arrhythmogenic cardiomyopathy (ACM; MIM #107970) is a primitive heart muscle disorder, characterized by the replacement of normal cardiomyocytes with fibrous or fibrofatty scar tissue [1], which eventually leads to the occurrence of ventricular arrhythmias [2]. Originally it was believed to involve only the right ventricle, which is why it was initially described as arrhythmogenic right ventricular cardiomyopathy (ARVC). Over time, it was observed that the right form represented only one variant of the disease [3]. Indeed, the most recent classification postulated three distinct categories of arrhythmogenic cardiomyopathy: the “right dominant” form, the “left dominant” form, and the “biventricular” form, depending on the distribution of the predominant morphofunctional abnormalities in the ventricular myocardium. The term “arrhythmogenic cardiomyopathy” better describes the wide spectrum of the phenotypic expression of the disease [4]. ACM with these characteristics is represented across several phenotypic categories in the 2023 European Society of Cardiology (ESC) Guidelines on Cardiomyopathies, highlighting potential overlaps particularly among the ARVC, dilated cardiomyopathy (DCM), and non-dilated left ventricular cardiomyopathy (NDLVC) phenotypes [5]. More recently, the Padua group proposed a new definition: “scarring/arrhythmogenic cardiomyopathy”, where not the localization but the presence of myocardial scarring defines this cardiomyopathy [6,7].

The incidence of the ACM varies throughout different regions, ranging from 1:1000 to 1:5000 in the general population, although it appears more common in regions such as the north-east of Italy [8]. There is a higher frequency of occurrence in men compared to women, with an approximate ratio of 3:1 [4].

Ventricular arrhythmias (VA) and sudden cardiac death (SCD) can be the first manifestation of the condition [8]. Fatal arrhythmic events can often occur in its early stages, whereas other patients may initially present with signs of heart failure (HF)—either right- or left-sided—depending on the predominant ventricular involvement [9]. In 2021, Bariani and colleagues observed that patients with ACM may also present with acute relapses of the disease, referred to as “hot phases” [10]. These represent acute stages of the illness, characterized by the presence of electrocardiographic abnormalities, the release of myocardial necrosis markers, and chest discomfort in the absence of coronary artery disease [11,12]. The inflammatory process appears to be a trigger for eventual tissue necrosis and replacement by fibroadipose tissue; nevertheless, the precise pathogenetic mechanism remains unknown [11,12].

In almost half of the cases, especially with a positive family history, a genetic etiology can be found [8]. Currently, mutations in more than 25 different genes have been described. In most cases, a heterozygous loss-of-function variant leads to haploinsufficiency and to an autosomal dominant modality of inheritance, though with incomplete penetrance and variable expressivity; rare autosomal recessive forms are described, where both alleles harbor a pathogenic variant [13]. The most relevant genes in ACM pathogenesis are the ones coding for junctional proteins, connecting cells exposed to nano-mechanical forces. Disfunctions in the desmosome, for example, have a major role in the pathogenesis of ACM, being *PKP2* (coding for plakophiln-2) the most commonly mutated gene in the disease. Plakoglobin (*JUP*) has a similar role, but mutations in this gene are rare and usually involved in recessive disorders. Plakophilin-2 and plakoglobin bind to the cytoplasmic domains of desmoglein-2 (*DSG2*) and desmocollin-2 (*DSC2*), transmembrane proteins from the cadherin family, and to desmoplakin (*DSP*) on the intracellular side, from the plakin cytolinker family. Other junctional genes recently associated with ACM are *CDH2* (encoding N-cadherin), *CTNNA3* (αT-catenin), *TJP1* (tight junction protein-1 or zonula occludens-1) and *ILK* (encoding the scaffolding adapter protein integrin linked kinase) [14]. In addition, other genes like *FLNC*, encoding the large cytolinker protein filamin-C, was already known for their correlation with other types of cardiomyopathies, but have been recently linked also to ACM [15]. A similar discourse can be made about *LMNA* (Lamin A/C), which is not a junctional gene, but can be included in the nuclear envelope proteins (together with minor ACM genes like *TMEM43* and *LEMD2*) and was historically associated with dilatative cardiomyopathy [14,16].

Among non-junctional genes, there is evidence of involvement of genes coding for Z-band proteins, like *DES* (desmin), *LDB3* (cipher) and *ACTN2* (α-actinin-2) [17].

Finally, heterozygous pathogenic variants in genes related to cardiac electrophysiology and generally associated with other arrhythmic disorders, like *RYR2*, *SCN5A*, and *PLN* have also been identified in ACM patients [14].

Early diagnosis and risk stratification are essential to identify patients at risk who may benefit from timely therapeutic intervention. Biomarkers are objective biological measures that indicate disease and can be reliably and accurately assessed [18,19]. An ideal biomarker should demonstrate high specificity, accuracy, and reproducibility, be easy to obtain and cost-effective; to be clinically valuable, it must provide additional information beyond existing diagnostic tools and support decision-making in clinical practice [18,19,20]. One of the main limitations of currently available biomarkers in ACM is their limited specificity, which reduces their utility in guiding optimal clinical decisions for this patient population.

Overcoming these limitations could pave the way for biomarkers to serve as valuable diagnostic and prognostic tools in patients with suspected ACM, offering a promising new perspective for this condition. Their integration into clinical practice may enable earlier detection of the disease, thereby supporting prompt and tailored management, especially considering the significant risk of SCD, malignant VA, and progressive heart failure in this population.

The aim of this narrative review is to provide an overview of the current knowledge regarding potential and emerging biomarkers in ACM.

## 2. Current Diagnostic Criteria

**Figure 1:**
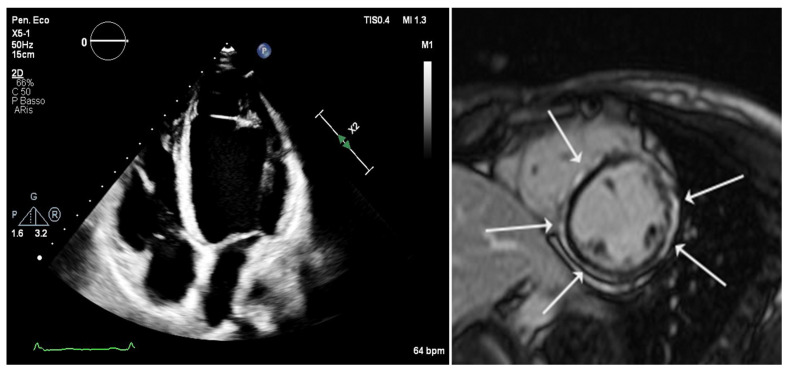
Multimodality cardiac imaging of left-dominant arrhythmogenic cardiomyopathy (LV-ACM) in a patient with a desmoplakin (DSP) variant. Left: Apical four-chamber view from a standard transthoracic echocardiogram demonstrating significant left ventricular (LV) dilation. Right: Corresponding short-axis late gadolinium enhancement (LGE) image from a cardiac magnetic resonance (CMR) scan confirming the underlying tissue pathology, showing a characteristic non-ischemic “ring-like” pattern of replacement fibrosis (hyperenhancement, white arrowheads), primarily localized to the subepicardial layer of the LV wall.

**Figure 2:**
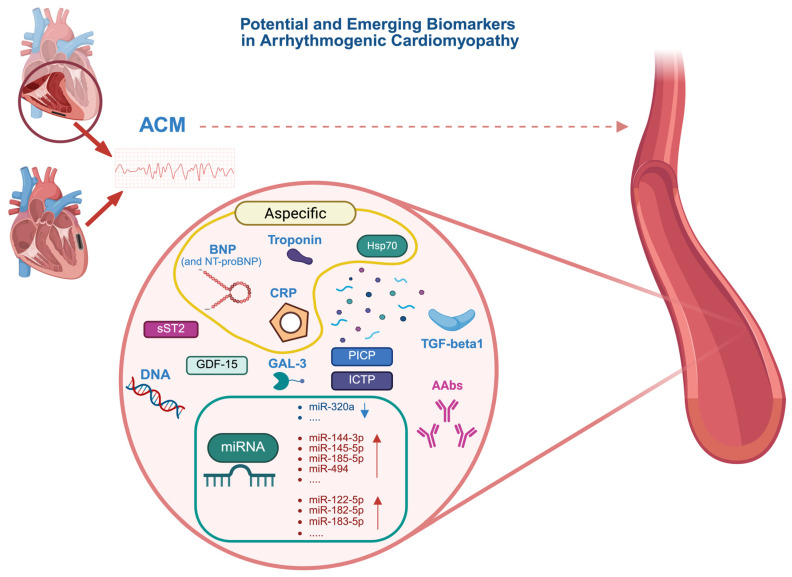
Potential and Emerging Biomarkers in Arrhythmogenic Cardiomyopathy (ACM). Blue downward arrows represent a reduction in biomarker levels in ACM, while red upward arrows indicate elevated levels observed in patients with ACM. AAbs, autoantibodies; ACM, arrhythmogenic cardiomyopathy; BNP, brain natriuretic peptide; CRP, c-reactive protein; DNA, deoxyribonucleic acid; GAL3, galectin-3; GDF-15, Growth Differentiation Factor 15; HSP70, heat shock protein 70; ICTP, cross-linked carboxy-terminal telopeptide of type I collagen; miRNA, microRNA (ribonucleic acid); NT-proBNP, N-terminal pro-B-type natriuretic peptide; PICP, carboxy-terminal propeptide of type I procollagen; sST2 soluble suppression of tumorigenicity-2; TGF-beta 1, transforming growth factor beta 1. This figure was created with BioRender.com.

The criteria for diagnosing right arrhythmogenic cardiomyopathy based solely on histopathological data were established by the 2010 task force guidelines [21,22]. The diagnosis of ACM relies on a scoring system that incorporates major and minor criteria [21]. The “Padua criteria” are newly proposed diagnostic standards for the identification of arrhythmogenic cardiomyopathy with left ventricular involvement, introduced in an international consensus statement in 2020 with specific diagnostic criteria [4]. Among the criteria outlined in the 2020 document, there is an update of the 2010 arrhythmogenic right ventricular cardiomyopathy criteria, incorporating tissue characterization through contrast-enhanced cardiac magnetic resonance imaging (cMRI) for the detection of fibrotic and fibro-fatty myocardial replacement in both ventricles, thus also describing the biventricular form of ACM [4]. CMRI plays a crucial role in the accurate identification of myocardial tissue alterations, particularly those characterized by scarring, which defines the phenotypic expression of the disease; this is especially relevant for the detection of late gadolinium enhancement (LGE) [4], as shown in Figure 1. More recently, a further update was released following a European task force report, which reviewed and refined the Padua diagnostic criteria, resulting in an enhanced and internationally endorsed consensus document [23]. Despite the ongoing revision of the diagnostic criteria, currently including morpho-functional, electrocardiographic, and arrhythmic burden criteria as well as the assessment of genetic make-up, no biochemical diagnostic markers have been included in the diagnostic parameters. Indeed, the early identification of ACM remains particularly challenging due to the absence of a single universally accepted clinical standard for diagnosis and the overlap with different cardiac phenotypes. In addition, the lack of specific diagnostic tools, whether imaging techniques or circulating biomarkers, further underscores the urgent need for reliable markers in ACM. Nonetheless, ongoing advances in biomarker discovery offer promising opportunities to improve diagnostic accuracy and patient management in this complex disease. The biomarkers described in this review are illustrated in Figure 2.

## 3. “Damage, Congestion, and Inflammation”: Widespread, Easy-to-Use, but Lacking Specificity

Circulating brain natriuretic peptide (BNP) is a standard biomarker for assessing the presence and progression of HF and congestion [24]. In patients with ACM, BNP has demonstrated utility in evaluating right ventricular (RV) dysfunction, exhibiting an inverse correlation with RV ejection fraction [25]. Notably, plasma BNP levels are elevated in ACM patients compared to those with idiopathic ventricular tachycardia (IVT), facilitating differential diagnosis. Similarly, the N-terminal fragment of BNP, NT-proBNP, has been linked to RV dysfunction and ventricular volumes in ACM, a finding corroborated by multiple studies [26]. However, the widespread applicability of BNP and NT-proBNP in ACM is limited by their low specificity, given their association with numerous other HF-related cardiac conditions [27].

The use of troponin as a marker of myocardial injury remains limited due to its low specificity, as elevated levels can be observed in a wide range of cardiac conditions, including acute decompensated heart failure, myocarditis, and, most notably, ischemic heart disease [18]. It should be noted that increases in troponin values are typical of ‘hot phase’ episodes, a challenging clinical presentation of ACM, characterized by acute chest pain and elevated cardiac troponins in the absence of obstructive coronary artery disease [12].

More peculiar is the research on heat shock protein 70 (HSP70) levels, identified as a marker of myocardial damage [28]. A 2009 study from Wei et colleagues revealed that the HSP70 serum levels were increased in ARVC failing hearts compared with non-failing hearts [29]. However, like BNP and NTproBNP, the usefulness of HSP70 as a specific marker for ACM is limited because it is not exclusively found in this condition.

Concerning the more conventional markers of inflammation, in this study, by Bonny et al., patients diagnosed with ARVC exhibit notably higher circulating levels of the inflammatory biomarker C-reactive protein (CRP) compared to individuals with idiopathic ventricular tachycardia (IVT) [30]. This suggests a strong correlation between the occurrence of VA and an acute inflammatory response in these individuals and a possible role as a prognostic marker given the association with arrhythmic episodes. In addition, higher levels of proinflammatory cytokines (such as interleukin-1β, interleukin-6, tumor necrosis factor-α) are also found in the bloodstream of ACM patients compared to healthy individuals [31,32].

Table 1 presents biomarkers of congestion and inflammation and the corresponding studies that evaluated them.

## 4. “Fibrosis: Early Signs, Not Just Damage”

**Figure 3:**
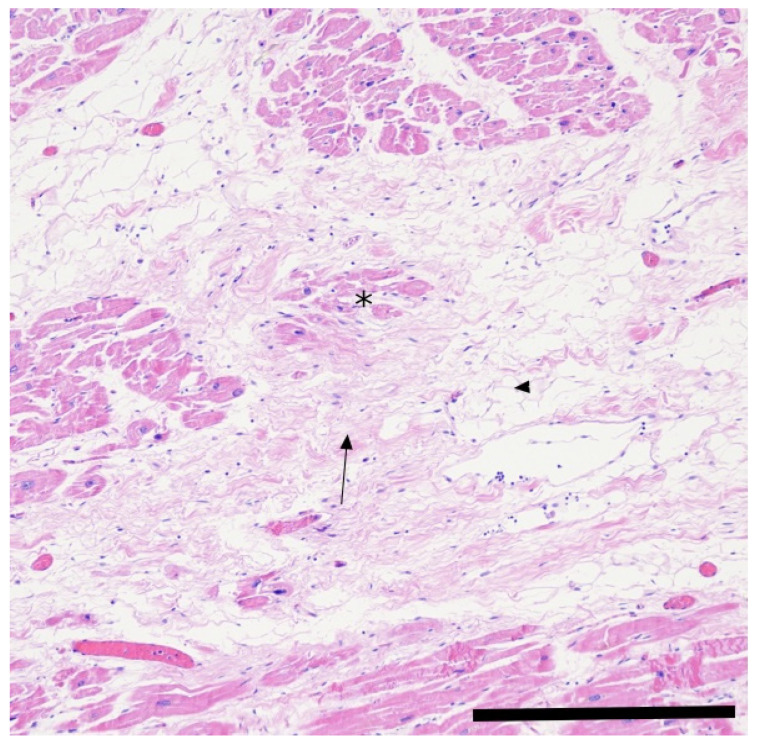
Histopathology. Medium-power histopathological view of an autopsy left ventricle from a 40-year-old male patient who had suddenly died from arrhythmogenic cardiomyopathy. This microscopic image shows a large fibro-fatty (arrow and arrowhead, respectively) myocardial replacement where some degenerating cardiomyocytes (asterisk) are entrapped. In addition, scattered chronic inflammatory cells are also present. Molecular analysis revealed a pathogenic variant in the first exon of the PKP2 gene. Staining: hematoxylin–eosin (×10 objective; bar is 400 μm).

Fibrosis is a common reparative response to cardiomyocyte injury in the heart. In ACM, fibrotic and fibro-fatty replacement of the myocardium—regardless of the affected ventricular region—typically progresses transmurally from the epicardium to the endocardium. Figure 3 illustrates a large fibro-fatty myocardial replacement. This process results in significant structural and functional alterations of the myocardial tissue and represents an important diagnostic feature, including the detection of LGE on cMRI [35,37].

In cardiac tissue, collagen type I and collagen type III are the predominant collagen types [41,42]. These collagen types are synthesized as pre-procollagen by fibroblasts. Procollagen is subsequently produced in the endoplasmic reticulum and then transferred to the extracellular matrix (ECM), where it undergoes enzymatic cleavage into amino (N)-propeptides and carboxy (C)-propeptides, typically in a 1:1:1 ratio. These propeptides are then released into the bloodstream. Furthermore, collagen type-I carboxy-terminal telopeptides (ICTP) are released into the bloodstream following the degradation of ECM constituents by matrix metalloproteinases (MMPs) [41].

As early as 2010, Kanoupakis and colleagues published a study involving 70 patients with non-ischemic dilated cardiomyopathy (NIDC). This research explored the utility of several fibrosis markers, including the C-terminal propeptide of collagen type-1, C-terminal telopeptide of collagen type-1, Matrix metalloproteinase-1, and Tissue inhibitor of matrix metalloproteinase-1, in predicting which patients would benefit from appropriate implantable cardioverter-defibrillator (ICD) therapies [43]. The results indicated that three of the four serologic markers were significantly higher in patients who received appropriate ICD therapy during follow-up compared to those who did not [43,44].

More recently, in 2022, van der Voorn et al. investigated the potential correlation between blood levels of procollagen type I carboxy-terminal propeptide (PICP), a marker of collagen synthesis, and the carboxy-terminal telopeptide of type I collagen (ICTP), a marker of collagen degradation, with disease severity in 72 patients (12 of whom had ACM) carrying the pathogenic phospholamban (PLN) p.Arg14del variant [33]. They observed weak to moderate correlations of these profibrotic biomarkers with QRS duration, ejection fraction (EF), end-diastolic volume (EDV), and end-systolic volume (ESV) in both the left and right ventricles. The study also revealed higher total collagen turnover, expressed as the PICP/ICTP ratio, in patients with premature ventricular contractions (PVCs) and T-wave inversions (TWI), particularly in leads V4–V6 [33].

In a separate study, van de Voorn et al. examined whether PICP and ICTP could serve as useful biomarkers for fibrosis formation in 45 patients, divided into 35 patients with ACM of various mutated genotypes, notably excluding patients with PLN variants, and 10 patients with preclinical variants [27]. Interestingly, they found higher plasma PICP levels and elevated total collagen turnover (PICP/ICTP ratio) in ACM-affected patients compared to preclinical variant carriers [27]. Furthermore, moderate to strong inverse correlations were observed between these biomarkers and the ejection fraction of both ventricles, indicating impaired contractile performance associated with pro-fibrotic remodeling [27].

Beyond collagen degradation products, other fibrosis-related molecules have been explored. Galectin-3 (GAL-3), a member of the galectin family of β-galactoside-binding lectins, is widely expressed in humans and plays crucial roles in various biological activities, including inflammation and fibrosis [45]. Myocardial injury triggers inflammatory and fibrotic signals that activate macrophages and stimulate GAL-3 secretion. Elevated serum levels of GAL-3 have been detected in nearly all types of cardiovascular diseases, and several studies have demonstrated its increasing levels in HF [46,47,48] and its role in the pathogenesis of ACM [49].

Oz et al. analyzed GAL-3 levels in patients with ARVC [34]. This observational study included 29 patients with ARVC and 24 healthy control individuals. Patients with ARVC exhibited significantly higher serum GAL-3 values. A cutoff value greater than 12.8 ng/mL demonstrated a 79% sensitivity and 76% specificity for predicting ARVC. Other findings suggested that left ventricular involvement, New York Heart Association (NYHA) functional class > 2 and GAL-3 levels were independent predictors of VA, with higher levels of these markers observed in patients experiencing ventricular tachycardia/ventricular fibrillation (VT/VF) episodes. GAL-3 levels were also elevated in patients with left ventricular involvement on cardiac magnetic resonance imaging and those with NYHA class > 2 [34].

The established correlation between the aforementioned markers with the severity of the arrhythmic events, specifically PVCs burden and VAs, indicates their potential utility for prognostic assessment. This suggests that these markers could be used to delineate arrhythmic risk and to determine the potential need for prophylactic measures to prevent sudden cardiac death.

Transforming Growth Factor-b1 (TGF-β1) is a central mediator of progressive myocardial fibrosis [50]. As a potent pro-fibrotic cytokine, it orchestrates the differentiation of cardiac fibroblasts into myofibroblasts, thereby instigating the excessive deposition of extracellular matrix proteins, notably collagen. Elevated circulating levels of TGF-β1 have been consistently documented across a spectrum of fibrotic cardiac conditions, indicating its potential utility as an early indicator of cardiac fibrosis [51]. A notable study by Maione et al. specifically assessed circulating levels of TGF-β1 in patients with ACM, revealing a statistically significant elevation in the ACM cohort compared to healthy controls [52]. This study further substantiated its findings by reporting increased fibrotic markers in cardiac biopsies obtained from ACM patients. These results collectively indicate a direct correlation between TGF-β1 elevated levels and the fibrotic processes characteristic of ACM [52]. This discovery holds significant clinical implications, as it provides evidence supporting the utility of TGF-β1 as a potential circulating biomarker for myocardial fibrosis in ACM. This builds upon existing mechanistic research on mice, which has established a link between defective desmosome adhesion in ACM and dysregulated integrin-αVβ6/TGF-β signaling, which subsequently contributes to cardiac fibrosis [53]. A further significant study on a transgenic DSC2 mice population showed profound changes in the expression of many genes involved in extracellular matrix (ECM) receptor interaction, cell adhesion and inflammatory response suggesting activation of cardiac fibrosis and remodeling processes [54].

Increased activation of the molecular cascades involving TGF was also confirmed in a more recent study [55], again carried out on mice carrying a specific variant of DSG2 that reproduced the same human variant identified in 49 individuals with ACM. The results of his study demonstrated a hyperactivation of the Activating Transcription Factor 4 (ATF4)/TGF-β1 signaling pathway as a potential mechanism driving progressive cardiac fibrosis in ACM [55].

The findings of these studies underscore the systemic involvement of TGF-β1 in the pathogenesis of ACM and its capacity to reflect underlying fibrotic remodeling [53,55], suggesting its potential utility as an additional diagnostic biomarker, indicative of fibrotic processes.

Recent work underscores the potential of spatial transcriptomic in identifying disease-related shifts in gene expression [56]. The study in question specifically documented an elevation in Zinc finger and BTB domain containing 11 (ZBTB11) activity among affected patients. This gene has been linked to the progression of cardiomyocyte atrophy and, in parallel, is correlated with the hyperactivation of the fibro-adipose replacement process [56]. The application of this method, therefore, represents a valuable tool for detecting the overexpression of genes associated with the fibrotic process. In the future, this finding could be the subject of further studies to evaluate its potential diagnostic application in clinical practice.

Fibrosis-related biomarkers investigated in ACM and the corresponding studies are summarized in Table 1.

## 5. “Novel Plasmatic Molecules”

### 5.1. Growth/Differentiation Factor-15 (GDF-15)

Growth/differentiation factor-15 (GDF-15) is a protein in the transforming growth factor-beta family [57]. Studies show a connection between GDF-15 levels and the presence of focal and diffuse fibrosis in the hearts of patients with HF, as detected by cMRI scans [58]. This link between the biomarker and the amount of myocardial fibrosis has also been confirmed through cardiac biopsies in people with advanced heart disease [57].

GDF-15 has emerged as a key marker for HF and other cardiovascular conditions. Elevated GDF-15 levels are associated with a greater likelihood of developing HF, including after a heart attack. It also reflects how severe the disease is and how it progresses, and importantly, it helps predict a patient’s outlook with HF [59]. The role of GDF15 as a biomarker has been analyzed in several studies involving patients with non-ischemic forms of dilated cardiomyopathy, showing a strong relationship with the fibrotic burden in the myocardium and a correlation with arrhythmic events and overall mortality [60,61]. However, the role of this molecule in ACM has been investigated by Akdis et al. in a study involving both a discovery and a validation cohort, comprising a total of 155 patients [36]. They analyzed the role of GDF-15, both individually and in combination with other biomarkers, as a predictor of ventricular involvement patterns and adverse outcomes in ACM [36]. In patients with ACM, those who also had left ventricular (LV) involvement showed elevated levels of GDF-15 compared to healthy individuals and patients with only right ventricular involvement [36]. GDF-15 levels were significantly associated with the presence of LGE on cMRI, a marker of myocardial scarring and fibrosis [36]. Notably, using a combination of three biomarkers—NT-proBNP, soluble suppression of tumorigenicity-2 (sST2), and GDF-15—proved most effective in predicting LV involvement in ACM patients [36].

### 5.2. Soluble Suppressor of Tumorigenicity-2

Suppression of tumorigenicity-2 (ST2) is a member of the interleukin-1 receptor family with two main forms, cellular (ST2L) and soluble (sST2), generated by alternative messenger ribonucleic acid (mRNA) processing [62]. The IL-33/ST2L signaling pathway is a mechanically activated cardioprotective system that exclusively uses the ST2L receptor. The circulating sST2 acts as a decoy receptor, binding to and sequestering IL-33, which antagonizes the cardioprotective effects of IL-33/ST2L interactions. Both cardiac fibroblasts and cardiomyocytes release sST2 in response to myocardial stress [63].

sST2 emerges as an independent indicator of prognosis in HF patients [62,63,64,65]. Its distinct secretion pathways suggest it could significantly enhance risk stratification [63]. However, current evidence largely comes from non-randomized studies, which is why it is not yet mentioned in recent ESC and American College of Cardiology (ACC)/American Heart Association (AHA) guidelines on HF management. The 2013 ACC/AHA guidelines did give a Class IIb recommendation for sST2 measurements for risk stratification and prognostication in chronic HF [62,66].

Three scientific studies have examined sST2 levels and their potential associations in patients with ACM. In line with findings on GDF-15, Akdis et al. reported elevated sST2 concentrations in ACM patients exhibiting biventricular involvement [36]. Combining NT-proBNP, sST2, and GDF-15 offers the most accurate prediction of left ventricular involvement in these patients [36]. Another study conducted by Borch et al. on 44 genotype-positive ACM patients found that sST2 levels were associated with RV global strain as well as with left ventricular (LV) function [37]. Elevated levels of sST2 were also independently associated with a history of VA [37]. The third study, performed by Borowiec and colleagues, involved 91 individuals with ACM and aimed to identify predictors of end-stage heart failure and assess the role of biomarkers in predicting adverse outcomes in ACM [38]. Elevated levels of sST2 (together with MMP-2, NT-proBNP, and troponin) were found to be associated with patients reaching the primary outcome of death or heart transplantation (HTx) [38]. However, these biomarkers did not prove useful in predicting the occurrence of VA [38].

With regard to the two markers discussed in the paragraph, they showed correlations with left ventricular involvement, LGE distribution, arrhythmic events and the need for transplantation, outlining a potential profile as prognostic indicators of disease severity.

The principal studies that evaluated GDF-15 and sST2 as biomarkers in ACM are presented in Table 1.

## 6. Autoimmunity Unveiling the Pathology

Several recent studies highlight the diagnostic potential of autoantibodies (AAbs) in ACM [67]. According to Chatterjee et al., the presence of autoantibodies against desmoglein-2 (DSG2) serves as a reliable and specific diagnostic marker for ACM [39]. These anti-DSG2 antibodies were detected in all 37 patients with definite ACM but were largely absent (in 31 out of 32) or only faintly present (in 1 out of 32) among control individuals [39]. However, the specificity of this marker for ACM, particularly in comparison to other cardiac diseases, still needs further investigation [67].

A study conducted by Caforio et al. investigated the presence of anti-heart autoantibodies (AHAs) and anti-intercalated disk autoantibodies (AIDAs) in patients with ARVC [40]. Using immunofluorescence microscopy, researchers observed a higher prevalence of both AHAs and AIDAs in ARVC probands (42 individuals) and their affected relatives (37 individuals) compared to patients with non-inflammatory cardiac disease, ischemic HF, and healthy controls [40]. This study also reported a correlation between the presence of these autoantibodies and indicators of disease severity in ARVC probands and affected relatives—for example, AHA-positive status was associated with a higher frequency of palpitations and with ICD implantation for the primary prevention of SCD, AIDA-positive status was associated with both lower RV and LV echocardiographic EF [39]. While these findings further support the presence of cardiac disease-specific autoantibodies in ACM, identifying their precise antigenic targets (epitopes) will be crucial for the development of reliable diagnostic tests.

Genetic mutations can lead to the formation of abnormally folded proteins or protein aggregates, which in turn may trigger an autoimmune response, resulting in the production of AAbs [66]. This suggests a potential future strategy for identifying cardiomyopathy-specific AAb profiles by focusing on proteins known to be abnormally expressed in certain genetic cardiomyopathies. For example, researchers could investigate AAbs targeting desmin and CRYAB-R102G protein aggregates in desminopathy [68,69], PLN-positive protein aggregates in PLN p.Arg14del cardiomyopathy [70], and TMEM43-positive protein aggregates in p.S358L TMEM43 ARVC [71].

The role of autoantibodies is a compelling area for advancement in medicine due to their specificity compared to traditional markers of organ damage, fibrosis, and inflammation. Their predominant utility is diagnostic, but emerging evidence also highlights their prognostic value, as their presence is associated with adverse outcomes such as arrhythmic events and reduced ejection fraction. This dual capability makes them a promising tool for both diagnosis and predicting a patient’s clinical trajectory.

An overview of the main studies assessing AAbs as biomarkers in ACM is shown in Table 1.

## 7. Genetic Testing and Familiar Predisposition

Constitutional genetic variants are not properly considered “biomarkers”, but since they may constitute an additional prognostic factor achievable through blood samples, few words should be spent on this topic [18]. Approximately 40-50% of patients with ACM, in fact, carry at least one pathogenic variant in genes encoding structural proteins, mostly PKP2, JUP, DSG2, DSC2, DSP, PLN, DES and FLNC [8,72]. There is also an important genetic and phenotypic overlap between ACM and DCM or, in a lesser extent, with other inherited cardiac diseases, especially concerning the heterogenous phenotypes related to LMNA gene variants [18,73]. Beside the well-known diagnostic value, for the proband and for family members, genetic testing on blood samples can also provide useful prognostic information. The European Society of Cardiology has published specific guidelines for cardiomyopathies in 2023 [5] remarking the importance of genetic testing also for risk stratification of sudden cardiac death. The most used risk calculators perform best in PKP2 positive patients [74], while the predictive accuracy is lower for DSP and PLN variants. Moreover, the majority of studies have focused on Caucasian populations, and data on less common ACM-associated genotypes in Western cohorts—such as DSG2, DSC2, and JUP—remain limited [75].

Considering the overlap with DCM, different studies showed that patients with LMNA pathogenic variants were at the highest risk of manifesting severe cardiac-related events, followed by DSP, PKP2 and FLNC. An Italian study attempted a multivariable analysis to properly identify the predictive value of genotype and phenotype, adjusting for familial forms, sex, age, and LVEF at baseline: ACM gene variants showed the strongest association with sudden cardiac death, heart transplant and major ventricular arrhythmia. The incidence of these events in DCM and ACM phenotypes was similar for patients carrying pathogenic variants in DSP, LMNA, and FLNC [76].

The practical interpretation of diagnostic genetic results may be difficult, with many variants being classified as Variants of Uncertain Significance (VoUS) or relatively frequent in the general population. A recent study based on a very large prospective cohort (United Kingdom Biobank, UKBB) confirmed the high frequency of pathogenic variants in ACM genes in the general populations and examined their potential consequences on health [77]. Of 200,619 UKBB participants, 5292 (2.64%) carried at least one predicted deleterious variant in cardiomyopathy-associated genes, including 767 (0.38%) patients with classic ARVC-related variants [77]. The presence of a predicted deleterious variant was significantly linked to increased all-cause mortality, which was predominantly driven by the sub-cohort with genes associated with DCM. Considering only ACM genes, the difference in terms of mortality was not significant, but data showed a higher risk of developing cardiomyopathy and composite outcomes (which included VA, HF, atrial fibrillation and stroke) [77].

Other important challenges in implementing genetic testing in clinical practice include incomplete and age-related penetrance, as well as variable expressivity. These aspects have been explored through genome-wide association studies (GWAS); however, the path towards identifying robust genetic modifiers and developing reliable polygenic risk scores remains long and complex [78,79].

## 8. The “miRNA Era”?

MicroRNAs (miRNAs) are small, non-coding RNAs (approximately 19 to 25 nucleotides long) that play a pivotal role in regulating gene expression at the post-transcriptional level by inhibiting translation or promoting the degradation of target messenger RNAs (mRNAs) [80,81,82]. MiRNAs are involved in various cellular processes, including cell cycle regulation, differentiation, apoptosis, fibrosis, and stress responses [67]. In the heart, miRNAs modulate pathways related to electrical conduction [83], cardiomyocyte survival [84], extracellular matrix remodeling [85] and intercellular adhesion structures [86]. Interestingly, all these mechanisms are disrupted in ACM. MiRNAs have recently emerged as promising diagnostic and therapeutic biomarkers in cardiology, given their stability in extracellular environments, including plasma and serum, and their ability to reflect disease-specific molecular changes [68]. Several miRNAs have been found to be dysregulated in ACM patients, suggesting their involvement in the molecular pathogenesis of the disease.

Sommariva et al. investigated circulating miRNAs in a cohort of 110 male subjects, including 36 patients with ACM, 21 with idiopathic ventricular tachycardia (IVT), and 53 healthy controls [86]. They observed significantly lower plasma levels of circulating miR-320a in patients with ACM compared to both healthy controls and individuals with IVT, supporting a potential role for this miRNA in disease pathogenesis and suggesting its possible utility in distinguishing ACM from IVT [86]. However, in this study, miR-320a expression did not correlate with disease severity [86].

Yamada et al. expanded upon previous research by screening 84 microRNAs—known to be associated with cardiac diseases—in a cohort of 62 patients presenting with VA [87]. Patients with definite ARVC showed significantly elevated plasma concentrations of miR-144-3p, miR-145-5p, miR-185-5p, and miR-494 compared to the other groups. Notably, higher plasma levels of miR-494 were associated with an increased risk of VA recurrence following catheter ablation in patients with definite ARVC [87].

Sacchetto et al. screened 754 circulating microRNAs in plasma samples from 21 patients with ARVC and 20 healthy controls, identifying a significant overexpression of miR-185-5p in affected individuals [88]. These findings support its potential utility as a circulating biomarker for ARVC. Moreover, the authors highlighted the potential involvement of miR-185-5p in regulating cell adhesion and modulating the Wnt and Hippo signaling pathways—mechanisms that are closely linked to ACM pathophysiology [88].

Bueno Marinas et al. conducted an integrated analysis of myocardial tissue and plasma samples, identifying a six-microRNA signature that demonstrated high discriminatory power in distinguishing ACM patients from healthy individuals and from those with other cardiomyopathies, including dilated, hypertrophic, and inflammatory forms, with area under the curve (AUC) values reaching up to 0.995 [89]. Specifically, miR-122-5p, miR-182-5p, and miR-183-5p were found to be upregulated in the plasma of ACM patients, while miR-133a-3p, miR-133b, and miR-142-3p were downregulated. The presence of this unique six-miRNA panel in both myocardial tissue and peripheral blood further supports its potential role in disease pathogenesis and its utility as a non-invasive biomarker for the diagnosis of ACM [89].

Beyond their diagnostic applications, certain miRNAs may also hold prognostic value. For instance, miR-494 has been associated not only with disease diagnosis but also with clinical outcomes, as its increased expression was found to predict a higher incidence of recurrent VA following catheter ablation procedures [87]. Similarly, elevated levels of miR-185-5p and miR-122-5p have been linked to more advanced disease phenotypes, including impaired ventricular function and a higher frequency of arrhythmic episodes [88,89]. These findings suggest that miRNAs could have both diagnostic and prognostic roles, contributing to risk stratification and, consequently, to personalized therapeutic planning. It is important to emphasize that these conclusions are based primarily on cross-sectional analyses or studies with small sample sizes. Longitudinal validation in larger, well-characterized cohorts is needed to confirm their predictive value over time.

MicroRNAs have also been analyzed in cardiac tissue specimens to explore their role at the myocardial level. In 2016, Zhang et al. conducted an analysis of the microRNA expression profile in cardiac tissue samples from 24 patients with ARVC, comparing these with samples from 24 healthy controls [90]. The study identified 21 microRNAs as distinctive signatures of ARVC, with 11 (including miR-21-5p) significantly upregulated and 10 (including miR-135b) significantly downregulated in ARVC myocardial tissue. Functional enrichment analysis revealed that miR-21-5p and miR-135b were associated with the Wnt and Hippo signaling pathways, suggesting their involvement in the molecular pathogenesis of ARVC [90]. Collectively, the findings supported a role for myocardial microRNAs in ARVC pathophysiology, with miR-21-5p and miR-135b possibly contributing to the fibro-fatty replacement of the myocardium and representing promising molecular targets in patients with ARVC [90].

In 2024, Bonet et al. analyzed four frozen RV myocardial biopsies from patients with ACM and four RV myocardial samples from individuals who had died from non-cardiac causes (used as controls) [80]. They found that three of the investigated microRNAs (miR-135a-5p, miR-140-3p, and miR-145-5p) were upregulated, while five (miR-486-5p, miR-486-3p, miR-125a-5p, let-7e-5p, and let-7d-3p) were downregulated in ACM heart samples [80].

Table 2 provides an overview of microRNAs investigated as biomarkers for ACM, along with the corresponding studies evaluating their diagnostic or prognostic value.

**Table 2:** Overview of studies investigating serum miRNA in patients with ACM.

Biomarkers	Type of Biomarkers	N. Patients with ACM	Main Findings in Affected Patients	Reference
**miR-320a**	miRNA (circulating)	36	miR-320a ↓ in ACM	Sommariva et al. [86]
**miR-144-3p, miR-145-5p, miR-185-5p, miR-494**	miRNA (circulating)	28 (definite ACM) 8 (borderline ACM) 3 (possible ACM)	miR-144-3p, 145-5p, 185-5p, and 494 were ↑ in patients with definite ARVC.	Yamada et al. [87]
**miR-185-5p**	miRNA (circulating)	37	miR-185-5p significantly ↑ in the plasma of ARVC patients	Sacchetto et al. [88]
**miR-122-5p, miR-182-5p, miR-183-5p, miR-133a-3p, miR-133b, miR-142-3p**	miRNA (myocardial sample and circulating)	106	miRNAs showed high discriminatory diagnostic power In plasma miR-122-5p, miR-182-5p, miR-183-5p were ↑ in ACM patients, while miR-133a-3p, miR-133b, and miR-142-3p were ↓ in ACM patients	Marinas et al. [89]
**miR-1183** **miR-29b-3p**	miRNA (myocardial sample)	8	Potential role of miR-29b-3p to ACM pathogenesis or phenotype maintenance	Rainer et al. [91]
**21 microRNAs as distinctive signatures of ARVC, in particular miR-21-5p and miR-135b**	miRNA (myocardial sample)	24	11 upregulated in ACM patients (including miR-21-5p) 10 downregulated in ACM patients (including miR-135b)	Zhang et al. [90]
**miR-135a-5p, miR-140-3p, miR-145-5p** **miR-486-5p, miR-486-3p, miR-125a-5p, let-7e-5p,** **let-7d-3p**	miRNA (myocardial sample)	4	miR-135a-5p, miR-140-3p, miR-145-5p were ↑ in ACM patients miR-486-5p, miR-486-3p, miR-125a-5p, let-7e-5p, let-7d-3p were ↓ in ACM patients	Bonet et. al. [80]

ACM, arrhythmogenic cardiomyopathy; ARVC, arrhythmogenic right ventricular cardiomyopathy; miRNA, microRNA (ribonucleic acid).

## 9. Clinical Applications, Limitations, and Future Directions

The clinical implementation of the majority of these novel biomarkers remains in its nascent stages. Although canonical markers of heart failure, such as BNP and NT-proBNP, and myocardial injury (troponin) are now routinely employed in most hospitals due to their low cost and accessibility, their integration into the standard assessment of ACM is not yet common practice. The same is true for markers of inflammation. Regarding more recent biomarkers—such as sST2, galectin-3, PICP, ICTP, and specific autoantibodies—their clinical application is currently non-existent. This is a consequence of their limited incorporation into established diagnostic pathways as well as the significant constraints on accessibility and cost of dedicated serum assays, which are largely confined to research institutions.

Despite encouraging results, the clinical implementation of miRNAs in ACM is hindered by several critical limitations. First, technical variability in sample collection, RNA isolation, and quantification methods limit reproducibility. The lack of consensus on normalization strategies and the absence of universally accepted reference miRNAs further complicate standardization. Moreover, the studies conducted to date are limited by small sample sizes, heterogeneous patient populations, and a predominant focus on late-stage, phenotypically manifest disease. Therefore, while the utility of miRNAs in detecting subclinical or genotype-positive/phenotype-negative individuals remain uncertain, this area represents a promising avenue for future investigation. Notably, the miRNA signature described by Bueno Marinas et al. [89] and the overexpression of miR-185-5p reported by Sacchetto et al. [88] may serve as useful tools for early diagnosis. Indeed, their well-recognized potential compared to other biomarker molecules may represent an advantage that could mitigate current technical limitations and support their translation into clinical practice. However, all published studies involving human samples to date have identified miRNAs in patients already exhibiting overt ACM phenotypes, but not necessarily at an early or preclinical stage of the disease. Thus, current evidence provides limited support for the use of miRNAs as reliable biomarkers for the early diagnosis of ACM [18,85]. In this regard, future studies will be essential to further explore the potential role of miRNAs as diagnostic biomarkers. The expansion of patient enrollment in future studies could strengthen the evidence base for these biomarkers. It is hoped that this will drive down costs and increase the availability of serum assays, enabling their adoption beyond specialized research institutions into routine clinical practice.

## 10. Conclusions

The evolving understanding of arrhythmogenic cardiomyopathy (ACM) highlights its complex nature. Despite significant advancements in diagnostic criteria—now incorporating morpho-functional, electrocardiographic, arrhythmic, and genetic assessments—the absence of specific biochemical markers remains a critical gap.

Traditional biomarkers such as BNP, NT-proBNP, troponin, and inflammatory markers, while indicative of cardiac stress or inflammation, lack the specificity required for a definitive ACM diagnosis. Genetic testing offers crucial diagnostic and prognostic insights, particularly in patients with known pathogenic variants, but challenges persist regarding variants of uncertain significance, incomplete penetrance, and variable expressivity. MicroRNAs are emerging as promising tools, with several dysregulated miRNAs identified as potential diagnostic and prognostic markers; however, their widespread clinical adoption is hindered by technical variability, small sample sizes, and the need for longitudinal validation. Similarly, fibrosis-related markers—such as collagen turnover products (PICP, ICTP), GAL-3, and TGF-β1—provide valuable information on myocardial remodeling in ACM. Novel circulating molecules, including GDF-15 and sST2, have shown potential as indicators of disease severity and ventricular involvement, particularly when assessed in combination. Lastly, the study of autoantibodies, especially anti-DSG2, offers an intriguing avenue for highly specific diagnostic biomarkers and points to a possible autoimmune component in ACM pathogenesis.

Further research involving larger patient populations is essential to overcome current limitations, standardize methodologies, and validate these and other promising biomarkers to ultimately enhance early diagnosis, risk stratification, and personalized management of ACM. Future progress in this field is contingent upon significant research efforts within large, rigorously characterized patient cohorts to achieve the validation of these emergent biomarkers and the standardization of their respective methodologies. The overall objective should be a paradigm shift from the pursuit of a singular definitive marker to the development and implementation of a comprehensive biomarker panel. Such a multi-marker assay—integrating indicators of distinct pathophysiological processes such as fibrosis (e.g., GAL-3, PICP), myocyte injury, genetic predisposition, and immune activation (e.g., anti-DSG2 antibodies)—would provide a more holistic and dynamic characterization of an individual’s disease state. Of critical importance, the maximal clinical utility of this approach will be realized through its synergistic integration with advanced imaging modalities. Correlating a validated biomarker profile with quantitative data derived from cMRI—including the extent of fibro-fatty infiltration, ventricular function, and late gadolinium enhancement—can support the development of highly specific, multimodal diagnostic algorithms. his integrated approach holds the potential to significantly advance the management of ACM by enhancing early diagnostic accuracy, improving risk stratification for sudden cardiac death, and supporting personalized therapeutic decisions.

## Figures and Tables

**Table 1:** Overview of studies evaluating biomarkers in patients with ACM (excluding microRNAs, which are presented separately in Table 2).

Biomarkers	Type of Biomarkers	N. Patients with ACM	Main Findings in Affected Patients	Reference
**BNP**	Congestion and HF	17	BNP ↑	Matsuo et al. [25]
**NT-proBNP**	Congestion and HF	56	NT-proBNP ↑ in patients with RV dysfunction	Cheng et al. [26]
**HSP70**	Myocardial damage	8	Tissue HSP70 ↑ (1.64-fold) in ARVC with HF HSP70 ↑ in serum samples of ARVC with HF	Wei et al. [29]
**CRP**	Inflammation	60	CRP ↑ in ARVC patients CRP ↑ within 24 h of VT episodes	Bonny et al. [30]
**PICP,** **ICTP,** **PICP/ICTP ratio**	Fibrosis	12	PICP/ICTP ratio moderately correlated with LV and RV EDV/ESV	van der Voorn et al. [33]
**PICP,** **ICTP,** **PICP/ICTP ratio**	Fibrosis	35	PICP/ICTP ratio ↑ in ACM PICP ↑ in ACM	van der Voorn et al. [27]
**Galectin-3**	Fibrosis	29	GAL3 ↑ in ARVC Independently predicted VT/VF episodes	Oz et al. [34]
**TGF-beta 1**	Fibrosis	52	TGF-beta 1 ↑ in ACM	Maione et al. [35]
**GDF-15** **sST2**	Novel biomarkers	108 (discovery cohort) 47 (validation cohort)	GDF-15 ↑ in LV involvement GDF-15 correlates with LGE GDF-15 + sST2 + NT-proBNP predicts BiV involvement sST2 ↑ in LV involvement sST2 correlates with LGE sST2 + GDF-15 + NT-proBNP predicts BiV involvement	Akdis et al. [36]
**sST2**	Novel biomarkers	44	sST2 ↑ correlates with: RV and LV dysfunction, VT	Broch et al. [37]
**sST2**	Novel biomarkers	91	sST2 ↑ prognostic factor of death or HTx	Borowiec et al. [38]
**Anti-DSG2**	Autoantibodies	20 (original cohort) 25 (validation cohort)	Anti-DSG2 was present in all 37 patients with definite ARVC Antibody density at Western blot or ELISA correlates with disease severity	Chatterjee et al. [39]
**AHAs** **+** **AIDAs**	Autoantibodies	42 ARVC + 37 ARs + 96 HRs	AHAs and AIDAs ↑ in ARVC, ARs, and HRs than the control population In ARVC and ARs, they were associated with features of disease severity (lower RVEF and LVEF)	Caforio et al. [40]

ACM, arrhythmogenic cardiomyopathy; AHAs, anti-heart autoantibodies; AIDAs, anti-intercalated disk autoantibodies; ARs, affected relatives; ARVC, arrhythmogenic right ventricular cardiomyopathy; BNP, brain natriuretic peptide; CRP, c-reactive protein; DSG2, desmoglein-2; EDV, end-diastolic volume; ESV, end-systolic volume; ESVi, end-systolic volume index; GAL3, galectin-3; GDF-15, Growth Differentiation Factor 15; HRs, healthy relatives; HSP70, heat shock protein 70; HTx, heart transplant; ICTP, collagen type-I carboxy-terminal telopeptide; LGE late gadolinium enhancement; LV, left ventricle; LVEF, left ventricular ejection fraction; NT-proBNP, N-terminal pro-B-type natriuretic peptide; PICP, carboxy-terminal propeptide of type I procollagen; RV, right ventricle; RVEF, right ventricular ejection fraction; sST2 soluble suppression of tumorigenicity-2; TGF-b1, transforming growth factor beta 1; VT, ventricular tachycardia.

## Data Availability

No new data were created or analyzed in this study.

## Abbreviations

The following abbreviations are used in this manuscript:

AAbs	Autoantibodies
ACM	Arrhythmogenic Cardiomyopathy
AHA	Anti-Heart Autoantibodies
AIDAs	Anti-Intercalated Disk Autoantibodies
ARVC	Arrhythmogenic Right Ventricular Cardiomyopathy
ATF4	Activating Transcription Factor 4
AUC	Area Under the Curve
BNP	Brain Natriuretic Peptide
cMRI	Cardiac Magnetic Resonance Imaging
CRP	C-Reactive Protein
DCM	Dilated Cardiomyopathy
DNA	Deoxyribonucleic Acid
DSC2	Desmocollin-2
DSG2	Desmoglein-2
DSP	Desmoplakin
ECM	Extracellular Matrix
EDV	End-Diastolic Volume
ESC	European Society of Cardiology
ESV	End-Systolic Volume
FLNC	Filamin-C
GAL3	Galectin-3
GDF-15	Growth Differentiation Factor 15
GWAS	Genome-Wide Association Studies
HC	Healthy Control
HCM	Hypertrophic Cardiomyopathy
HF	Heart Failure
HSP70	Heat Shock Protein 70
HTx	Heart Transplantation
ICD	Implantable Cardioverter Defibrillator
ICTP	Collagen Type-I Carboxy-Terminal Telopeptide
IHD	Ischemic Heart Disease
IVT	Idiopathic Ventricular Tachycardia
JUP	Plakoglobin
LGE	Late Gadolinium Enhancement
LMNA	Lamin A/C
LV	Left Ventricle
LVEF	Left Ventricular Ejection Fraction
miRNA	MicroRNA
MMPs	Matrix Metalloproteinases
mRNA	messenger RNA
NDLVC	Non-Dilated Left Ventricular Cardiomyopathy
NICD	Noninflammatory Cardiac Disease
NT-proBNP	N-terminal pro-B-type natriuretic peptide
NYHA	New York Heart Association
PICP	Procollagen Type-I Carboxy-Terminal Propeptides
PKP2	Plakophilin-2
PLN	Phospholamban
PVCs	Premature Ventricular Contractions
RNA	Ribonucleic Acid
RV	Right Ventricle
RVEF	Right Ventricular Ejection Fraction
RVOT	Right Ventricular Outflow Tract
RYR2	Ryanodine-2
SCD	Sudden Cardiac Death
SCN5A	Sodium Voltage-gated Channel Alpha subunit 5
sST2	Soluble Suppression of Tumorigenicity 2
TGF-β1	Transforming Growth Factor Beta 1
TMEM43	Transmembrane protein-43
TTN	Titin
TWI	T wave inversion
UKBB	United Kingdom Biobank
VA	Ventricular Arrhythmias
VoUS	Variants of Uncertain Significance
VF	Ventricular Fibrillation
VT	Ventricular Tachycardia
